# Types of Racism and Health Disparities and Inequalities among Cancer Patients: An Editorial Reflection of Articles in This Special Issue of *IJERPH*

**DOI:** 10.3390/ijerph21060785

**Published:** 2024-06-17

**Authors:** Shaila M. Strayhorn-Carter, Ken Batai, Francine C. Gachupin

**Affiliations:** 1 Department of Public Health, School of Health & Applied Human Sciences, University of North Carolina Wilmington, Wilmington, NC 28403, USA; strayhorns@uncw.edu; 2 Department of Cancer Prevention & Control, Roswell Park Comprehensive Cancer Center, Buffalo, NY 14203, USA; 3 Department of Family and Community Medicine, College of Medicine, University of Arizona, Tucson, AZ 85721, USA; fcgachupin@arizona.edu

## Abstract

Racism has been a long-standing influential factor that has negatively impacted both past and current health disparities within the United Sates population. Existing problems of racism and its impact on both health disparities and health inequalities were only amplified during the COVID-19 pandemic. The pandemic allowed both clinicians and researchers to recognize a growing list of health concerns at the macro-, meso-, and micro-level among underserved racially minoritized patients with specific chronic illnesses such as cancer. Based on these concerns, this Special Issue was designed to highlight the challenges of cancer screening, cancer treatment, and cancer-centered educational outreach among racially minoritized communities.

## 1. Introduction

“It is impossible to heal in a space that will not acknowledge or discuss racism” [1] is a quote by a woman of color who noted that an individual’s health can be directly impacted by racism. Recent events that range from social injustices and police brutality to the COVID-19 pandemic have shed light on the relationship between racism and both health disparities and inequalities. Moreover, recent evidence of blatant racism has made healthcare professionals and politicians increasingly aware of the widening gap in health disparities and inequalities among patients with chronic conditions such as cancer. A recent report observed that various types of racism may have contributed to countless cancer-related deaths among patients from minoritized racial and ethnic groups [2]. However, more information is needed to understand how these various types of racism may be linked to both health inequalities and health disparities among cancer survivors.

### 1.1. What Is Racism?

There are many definitions that scholars use to define racism. A popular definition is that it is a position of both superiority and inferiority among humanity that is centered on economic, political, and cultural differences [3]. This definition suggests that racism can be linked to socioeconomic factors. However, the color or race of an individual’s skin is a prominent factor of racism. Racism has been a consistent practice throughout the United States (U.S.) since first contact in 1492 for American Indians [4] and the 1600s for others as a means of treating individuals as inferior due to the expression of their skin pigmentation [5]. Racism can be observed among those who consider themselves to be racially superior to those of a different skin color and choose to believe that their own physical traits (i.e., hair texture, shape of nose, and eye color) are ideal [6], which in turn can lead to these individuals harboring negative feelings and thoughts towards a certain race [7,8]. In addition to this, specific types of racism can also lead to prejudices and stereotypes among racially and ethnically minoritized populations [9,10,11].

### 1.2. Types of Racism

Racism is a multi-level concept and is often categorized into a macro-, meso-, and micro-level. Here, we describe each level of racism using examples of racism in the U.S.

Macro-level: Macro-level or structural racism focuses on the oppression of minoritized groups due to the implementation of federal and state-level policies and laws [12,13]. Previous examples of macro-level racism in the U.S. have been observed with the placement of American Indians on reservations [14] and in boarding schools [15], within the implementation of Jim Crow laws targeting non-Hispanic Black communities [16], the discriminatory efforts of Voting Rights Act towards people of color [17], anti-immigration acts such as the Chinese Exclusion Act of 1882 and Immigration Act of 1924 [18], and the Executive Order 9066 which forcibly removed individuals of Japanese ancestry from their homes in the U.S [19]. However, macro-level racism continues to be an apparent event today. For example, bank lending and home ownership policies have historically been more advantageous for non-Hispanic White individuals compared to minoritized populations, and such practices are continuously observed [10,20,21]. Anti-immigration rhetoric continues to dominate in political and public discourse [22].

Meso-level: Meso-level racism (also known as institutional racism) is racism found within education, healthcare, and governmental systems [23]. News reports and social media have drawn attention to racial inequalities within the justice system, specifically in the form of police brutality. Deaths of innocent racially minoritized groups have been on the rise due to the population being perceived as criminals because of their physical appearance. George Floyd, Breonna Taylor, and Brandon Laducer are just a few of the innocent victims whose lives were cut short because of police brutality. Abuse of authority has been associated with medical mistrust in the form of various unmet medical needs within the healthcare system [24,25].

Racism in the healthcare system not only increases the unmet needs of patients from minoritized groups, as described in a landmark publication from the Institute of Medicine [26], but has also increased the likelihood of the onset of various chronic diseases [27,28]. Disproportional increases in diseases in marginalized communities are also an example of health disparities and health inequalities. Health disparities are driven by social, economic, and environmental disadvantages linked to discriminatory factors such as race [29]. Alternatively, health inequalities focus on differences in health statuses among individuals based on their social, economic, demographic, and geographic backgrounds [30]. These concepts can directly impact both interpersonal- and intrapersonal-level factors of racism.

Micro-level: Micro-level racism refers to both interpersonal and internalized racism. Interpersonal-level racism refers to discrimination between various racial, ethical, and cultural groups [31]. This form of racism can manifest itself as both explicit and implicit biases towards a racial group(s) [32]. Ultimately, it is peer-to-peer social relationship that largely influences this type of racism in social relationships [33]. Such examples of interpersonal racism can include—but are not limited to—acts of discrimination, name-calling, and unequal treatment/shunning behaviors towards another race. Another type of micro-level racism is intrapersonal or internalized racism. Internalized racism is based on the belief among marginalized racial groups that their oppression by dominant racial groups should be accepted or considered “normal” [34,35]. Internalized racism is often a response to experiences of macro- and meso-level racism that an individual may face. When dealing with racism at the macro- or meso-level, individuals of marginalized racial communities may choose to internalize their negative feelings by not responding or ignoring acts of discrimination [36]. Such a method of internalization of racism has been shown to increase stress and psychological problems among minoritized groups [37]. Furthermore, the internalization of racism can not only lead to stress but has been identified as a potential socially driving factor for chronic diseases such as cardiovascular disease [38], diabetes [39], and cancer [40].

### 1.3. Racism and the Pandemic

The types of racism mentioned above became more apparent both during and after the COVID-19 pandemic. According to a study conducted by the Pew Research Center, non-Hispanic Black and Asian individuals were more likely to encounter negative experiences due to their race compared to other racial groups during the pandemic [41]. Individuals of Asian descent reported experiencing increased hate crimes during the pandemic due to political figures mislabeling the virus in a manner that stigmatized members of the Asian communities [42]. Racial slurs, hate speech, obscene gestures, and graffiti are just a few of the examples of hate crimes experienced within these communities [43].

Increased experiences of racism were also apparent within other racial communities. A recent study surveyed minoritized groups in October 2020 to assess the association between perceived racism and negative mental health outcomes [44]. It was found that perceived racism was the highest among participants who identified as non-Hispanic Black. The study also identified that increased experiences of racism online and through social media platforms were common among participants who self-identified as Hispanic, non-Hispanic Black, East Asian, South Asian, and Southeast Asian. These findings coincide with previous reports of the rise in racism among minoritized groups, which in turn can lead to negative health outcomes. For example, the pandemic exacerbated existing health disparities for American Indians and Alaska Natives, as they were more likely to be infected, experience complications, and die from COVID-19 [45]. In fact, life expectancy for American Indian and Alaska Natives decreased by 6.4 years between 2019 and 2021 [46].

### 1.4. Racism, the Pandemic, and Cancer

Alongside these increased experiences of racism, there have been significant delays in cancer care among patients from minoritized populations [47]. Cancer-related death rates among minoritized groups were already exceptionally high prior to the pandemic [48]. However, the pandemic resulted in significant delays in both cancer prevention [49,50] and cancer treatment [51] across the general population. These delays, coupled with varying experiences of racism, may have increased the growing trends of cancer-related death rates among certain racially minoritized populations. The percentage of cancer death with COVID-19 in 2021 was higher in American Indian/Alaska Native (3.4%), Latino/a (3.2%), and non-Hispanic Black (2.5%) people compared to other groups (ranging between 1.5% and 2.3%) [52].

U.S. federal regulations restricting citizenship rights were passed during the pandemic period. These restrictions made it a challenge for certain members of Latino communities to obtain access to health insurance, thus inhibiting their ability to receive cancer screenings and cancer treatments [53]. Low rates of cancer screenings have been documented among members of the Latino/a [54], Asian [55,56], non-Hispanic Black [55], and Native Hawaiian/Pacific Islander communities when compared to the non-Hispanic White community [57]. Experiences of macro-, meso-, and micro-level racism during the pandemic may have increased these health disparities and health inequalities.

The pandemic not only brought awareness to existing health disparities and inequalities within minoritized communities but also altered the quality of cancer care among members of these communities as well. A recent study conducted by Patel et al. assessed the relationship between race and ethnicity among patients with cancer and the primary outcomes of cancer treatment delays. Their findings revealed that non-Hispanic Black cancer patients had three times greater odds of experiencing a treatment delay of up to four weeks compared to non-Hispanic White cancer patients [58]. Similarly, both non-Hispanic Black and Latino/a cancer patients reported higher odds of experiencing modifications in their cancer treatments which led to them fearing these changes could worsen their cancer outcomes [58]. Another study by Llanos et al. also found that non-Hispanic Black cancer patients with SARS-CoV-2 infection were more likely to delay or discontinue cancer treatment [59].

While many factors may have impacted the quality of cancer care assessed within studies [58,59], varying forms of racism continue to be one of the strongest factors affecting the growing health disparities and health inequalities among minoritized populations. The pandemic made it clear that the widening gap in health disparities and health inequality will only continue if racism within our country is not addressed. Failure to do so will only result in an increased gap in cancer mortality rates and a greater challenge in cancer care among minoritized populations.

## 2. Articles in This Special Issue

This editorial will focus on providing insight into the relationship between types of racism and both health inequalities and health disparities among cancer patients from diverse backgrounds. The papers included in the previous and current Special Issue on cancer health disparities and public health address various aspects of cancer health disparities derived from structural inequality and racism. Frameworks, evidence of disparities, challenges that racially and ethnically minoritized communities face, gaps in our knowledge, and community-based approaches related to disparities in cancer care are presented in these papers. Below are brief summaries of a few of the articles available in this second Special Issue.

Five articles within this Special Issue focus on cancer screening among racially minoritized populations. Smayda et al., in their paper titled “Cancer Screening Prevalence among Participants in the Southcentral Alaska Education and Research towards Health (EARTH) Study at Baseline and Follow-Up” [60], assess the prevalence of self-reported colorectal, cervical, and breast cancer screening among Alaska Native communities. They discover dramatic declines in specific cancer screening behaviors within this population over a 10-year follow-up.

The next article, by Taylor et al., is titled ““A Huge Gap”: Health Care Provider Perspectives on Cancer Screening for Aboriginal and Torres Strait Islander People in the Northern Territory” [61]. The authors were able to conduct qualitative research on the perspectives and approaches of primary care physicians who screen both Aboriginal and Torres Strait Islander people in the Australian Northern Territory for various types of cancers. Their findings emphasize the importance of designing culturally targeted educational outreach platforms to better reduce the cancer burden within this community.

The authors of “Improving Guideline-Recommended Colorectal Cancer Screening in a Federally Qualified Health Center (FQHC): Implementing a Patient Navigation and Practice Facilitation Intervention to Promote Health Equity” focus on addressing barriers to colorectal cancer screening within an urban FQHC [62], while also exploring the effectiveness of early cancer detection within populations with limited English proficiency. Through their findings, the authors hope to increase colorectal cancer screening among this underserved population.

The fourth article in this series, “Mi-CARE: Comparing Three Evidence-Based Interventions to Promote Colorectal Cancer Screening among Ethnic Minorities within Three Different Clinical Contexts” also discusses improving colorectal cancer screening within an FQHC [63]. Specifically, the authors assess the effectiveness of an evidence-based intervention designed to improve colorectal cancer screening among individuals from racially and ethnically minoritized communities who identify as either non-Hispanic Black or Latino/a. The researchers hope that their findings will encourage additional clinics to proactively help minoritized populations consistently attend colorectal cancer screenings.

Finally, “Provider- and System-Level Barriers and Facilitators to Colonoscopy and Multi-Target Stool DNA for Colorectal Cancer Screening in Rural/Remote Alaska Native Communities” conducts a qualitative analysis among providers serving Alaska Native communities [64]. With this qualitative dataset as a starting point, the study identifies and explores themes focusing on various factors that either hinder or facilitate colorectal cancer screening within these communities.

Two articles focus on community-centered approaches to improve the health of participants through community partnerships. Molina et al. present a manuscript titled “Equity in Cancer and Chronic Disease Prevention through a Multi-Pronged Network Intervention: Works-in-Progress” which focuses on improving health literacy and information transfer to prevent chronic diseases such as cancer among a racially diverse population in Chicago, IL [65]. Chicago residents who participated in the Community Health Response Corps Program can connect to local health resources. Through this program, the authors develop a community-centered model and resources to assist residents in the greater Chicago area in becoming advocates for disease prevention.

The second article, by Tossas et al., is titled “The Chickahominy T.R.U.T.H. (Trust, Research, Understand, Teach, and Heal) Project—A Tribal Community–Academic Partnership for Understanding the Impact of Structural Factors on Perceived Cancer Risk in Rural Virginia” [66]. The authors describe a community and academic partnership to identify structural- and individual-level factors that may contribute to cancer risk among high-risk populations. The findings enable the authors to utilize a culturally tailored program to improve cancer education and resources among high-risk populations.

The remaining two articles in this *IJERPH* Special Issue identify strategies for improving cancer care through focus groups with participants from minoritized groups. The first of these two, “Medical Advocacy among Latina Women Diagnosed with Breast Cancer”, collected qualitative data among Latina breast cancer survivors to understand their experiences during their breast cancer journey [67]. The study found that community advocates facilitate Latina breast cancer survivors overcoming multiple barriers to care through education, peer support, and access to resources. The results suggest the importance of collaboration among researchers, clinicians, and community stakeholders to understand the specific healthcare needs of individuals within this population.

Lastly, Smith et al. present “An Exploration of Black Men’s Attitudes and Experiences Communicating with Dentists about Oral and Pharyngeal Cancer” [68]. The purpose of their study is to explore the attitudes and experiences of Black men when communicating with dentists about oral and pharyngeal cancer. The results aid dentists in improving patient–provider communication among members of this population.

## 3. Conclusions

As Guest Editors, we hope that the information presented in the previous and current Special Issues can help develop investigations to further understand the relationships between various types of racism and cancer disparities. In addition, the findings of the articles in these collections can be an invaluable source aiding in the development of recommendations, interventions, programs, and policies for building health equity. We would also like to thank all authors who contributed to this Special Issue.

## Data Availability

No new data were produced. Data sharing is not applicable to this article.
